# Phylogenetic Diversity of NTT Nucleotide Transport Proteins in Free-Living and Parasitic Bacteria and Eukaryotes

**DOI:** 10.1093/gbe/evx015

**Published:** 2017-02-02

**Authors:** Peter Major, T. Martin Embley, Tom A. Williams

**Affiliations:** 1 Institute for Cell and Molecular Biosciences, University of Newcastle, Newcastle upon Tyne, United Kingdom; 2 School of Earth Sciences, University of Bristol, United Kingdom

**Keywords:** nucleotide transport proteins, phylogenetics, parasitism, symbiosis

## Abstract

Plasma membrane-located nucleotide transport proteins (NTTs) underpin the lifestyle of important obligate intracellular bacterial and eukaryotic pathogens by importing energy and nucleotides from infected host cells that the pathogens can no longer make for themselves. As such their presence is often seen as a hallmark of an intracellular lifestyle associated with reductive genome evolution and loss of primary biosynthetic pathways. Here, we investigate the phylogenetic distribution of NTT sequences across the domains of cellular life. Our analysis reveals an unexpectedly broad distribution of NTT genes in both host-associated and free-living prokaryotes and eukaryotes. We also identify cases of within-bacteria and bacteria-to-eukaryote horizontal NTT transfer, including into the base of the oomycetes, a major clade of parasitic eukaryotes. In addition to identifying sequences that retain the canonical NTT structure, we detected NTT gene fusions with HEAT-repeat and cyclic nucleotide binding domains in Cyanobacteria, pathogenic *Chlamydiae* and Oomycetes. Our results suggest that NTTs are versatile functional modules with a much wider distribution and a broader range of potential roles than has previously been appreciated.

## Introduction

Nucleotides are essential biomolecules for every living organism. They are needed for DNA and RNA synthesis, and the nucleotide ATP is the universal cellular energy currency. ATP can be produced through photophosphorylation, oxidative phosphorylation, substrate-level phosphorylation, glycolysis, or through hydrolysis of arginine (Schimke et al. 1966; Allen 2002; Lunt and Vander Heiden 2011; Müller et al. 2012). *De novo* synthesis of nucleotides is energetically expensive: one nucleotide costs ∼50 ATP molecules (Lynch and Marinov 2015). As a result, intracellular parasites and symbionts have repeatedly and convergently evolved an alternative means of obtaining the nucleotides they need for growth and proliferation by importing them from the infected host cell. Intriguingly, intracellular parasites and pathogens from across the tree of life, including bacteria like *Chlamydia* and *Rickettsia* and eukaryotes such as the Microsporidia, a group of endoparasitic fungi, steal host energy using the same family of surface-located nucleotide transport proteins (NTTs), which they appear to share by horizontal gene transfer (Haferkamp et al. 2004, 2006; Audia and Winkler 2006; Tsaousis et al. 2008; Heinz et al. 2014). In the Microsporidia, acquisition of an ancestral NTT gene was an early step in the transition from a free-living to an obligately intracellular parasitic lifestyle, which subsequently led to the loss of many biosynthetic pathways and a drastic reduction in genome size and content (Nakjang et al. 2013; Heinz et al. 2014). NTT proteins are members of the AAA protein family (Winkler and Neuhaus, 1999), and all functionally characterized NTT proteins are from parasites with reduced genomes or from endosymbiotic organelles such as plastids, where their roles in the uptake of nucleotides, ATP and other host metabolites have been investigated in a series of articles (Kampfenkel et al. 1995; Neuhaus et al. 1997; Tjaden et al. 1998, 1999; Haferkamp et al. 2004, 2006; Schmitz-Esser et al. 2004, 2008; Audia and Winkler 2006; Tsaousis et al. 2008; Ast et al. 2009; Knab et al. 2011; Heinz et al. 2014; Chu et al. 2017). Here, we have investigated the phylogenetic distribution of NTT proteins across the tree of life and show that it is much broader than previously appreciated. Surprisingly, we find NTT genes conserved on the genomes of diverse free-living organisms and we also identify NTT domains in a variety of multidomain contexts in which the NTT domain forms part of a larger multifunctional whole.

## Widespread Distribution and Novel Functional Contexts for the NTT Domain

**Fig. 1.—:**
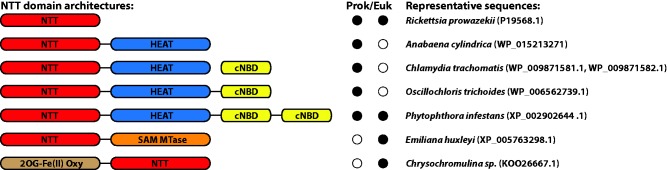
A diversity of NTT domain-containing protein architectures. In addition to canonical single-domain NTT proteins such as those used for ATP uptake by intracellular parasites, we identified five other multidomain protein architectures that include an NTT domain. The functions of these fusion genes are unknown, although gene fusion often occurs between genes or domains that interact to carry out a metabolic function (Enright et al. 1999). Examples of all of these architectures are found in free-living organisms, implying a diversity of NTT functions beyond their canonical roles in energy transfer in plastids and intracellular parasites. One clade of NTT fusion proteins (NTT-HEAT) is very widely conserved in bacteria, including in cyanobacteria, and is also found in oomycetes. SAM MTase: S-adenosyl-L-methionine-dependent methyltransferase; 2OG-Fe(II) oxygenase (Oxoglutarate/iron-dependent dioxygenase); cNBD: cyclic nucleotide binding domain.

To investigate the phylogenetic and functional diversity of NTTs, we searched sequenced genomes annotated at NCBI using BLASTP and extracted 377 sequences from 36 taxa for a phylogenetic analysis (table 1). In addition to proteins with the canonical single-domain NTT structure (PF03219 in the Pfam database) (Finn et al. 2014), we identified five additional gene architectures in which an NTT domain forms part of a larger whole (fig. 1). Two of the NTT architectures—NTT-SAM MTase and 2OG-Fe(II) Oxy-NTT (fig. 1)—are restricted to a single species, and so we cannot exclude the possibility that they are the result of database annotation errors; if genuine, they presumably arose from recent gene fusion events. However, another architecture—in which an NTT domain is C-terminally fused to a HEAT-repeat domain (PF13646 and PF02985; “Huntingtin, elongation factor 3, protein phosphatase 2A, TOR1” repeat, named after some of the proteins in which it appears), is very broadly conserved. In some cases, these NTT-HEAT fusions were immediately followed on the genome sequence by a cyclic nucleotide binding domain (cNBD; PF00027) at a distance of several nucleotides, suggesting that both proteins are part of the same operon and might be functionally linked. Consistent with this hypothesis, our searches also identified proteins in which this cNBD domain has become fused to the C-terminal end of the upstream protein, resulting in a three-domain NTT-HEAT-cNBD fusion. Fused NTTs of both kinds (NTT-HEAT or NTT-HEAT-cNBD) are found predominantly in bacteria. Although the functional interplay between these fused domains is currently unknown, HEAT repeats are known to be involved in protein-protein interactions (Groves et al. 1999). One possible role of a cNBD domain could be to bind cAMP produced from ATP by adenylate cyclase, potentially adjusting NTT-mediated ATP uptake to the energy requirements of the cell. In support of this idea, a molecular dynamics study on the HEAT-repeat protein PR65 of phosphatase PP2A revealed that conformational changes affect substrate binding and catalysis (Grinthal et al. 2010).

**Table 1:** Organisms and Corresponding Number of NTT Sequences Used in This Study

Taxon	Number of Sequences
Cyanobacteria	63
Alphaproteobacteria	33
Betaproteobacteria	7
Gammaproteobacteria	45
Deltaproteobacteria	33
Epsilonproteobacteria	3
Zetaproteobacteria	1
Chlamydiae	30
Bacteroidetes	43
Bacilli	5
Actinobacteria	1
Chloroflexi	2
Clostridia	1
Acidobacteria	3
Verrucomicrobia	2
Planctomycetes	2
Deinococcus-Thermus	1
Fibrobacteres	1
Gemmatimonadetes	2
Nitrospirae	1
Poribacteria	1
Marinimicrobia	1
Aminicenantes	1
Latescibacteria	1
Viridiplantae	23
Stramenopiles	32
Rhizaria	2
Alveolata	2
Rhodophyta	3
Glaucocystophyta	1
Cryptophyta	1
Haptophyceae	14
Euglenozoa	1
Ichthyosporea	2
Fungi	11
Apusozoa	2

Note.—No NTTs were found in Spirochaetes, Chlorobi, Aquificae, Dictyoglomi, Elusimicrobia, Fusobacteria, Tenericutes, Thermotogae, or any group of Archaea.

## Characterized Plastid and Parasite Transporters Represent Only a Small Subset of NTT Diversity

**Fig. 2.—:**
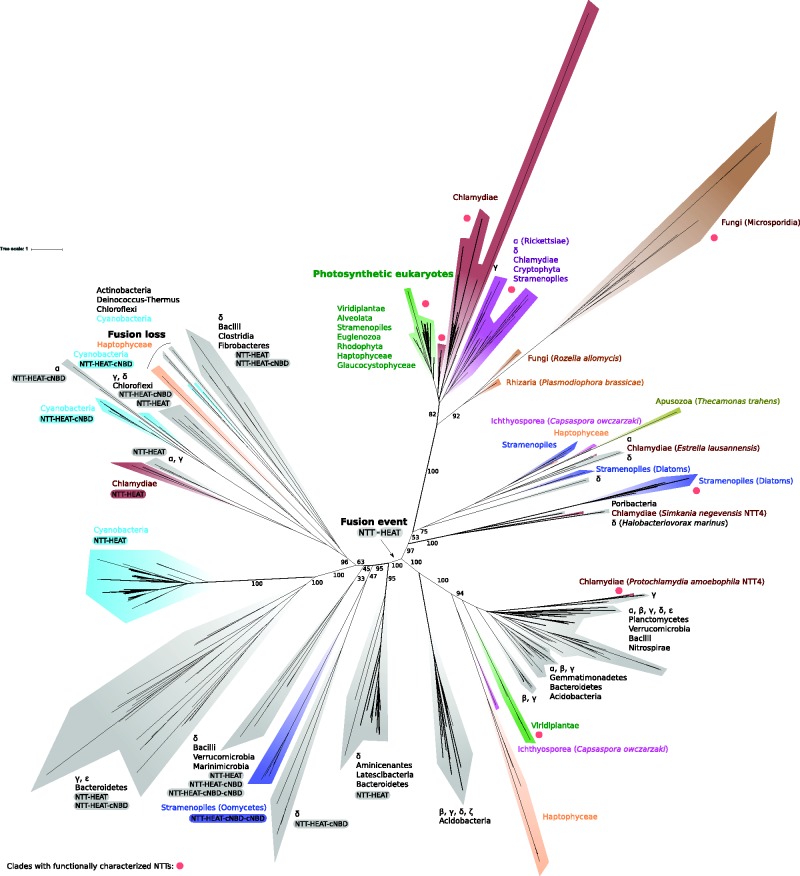
Phylogeny of NTT domain-containing proteins, highlighting the range of phylogenetic and protein structural contexts in which NTT domains are observed. Although the tree is unrooted, bacterial sequences group at the base of each of the major clans, suggesting a bacterial origin of the domain followed by horizontal transfers into eukaryotes. With the exception of three proteins (an NAD transporter from *P. amoebophila*, a purine transporter from *P. tricornutum*, and NTT3 of *A. thaliana*), all experimentally characterized NTTs from both bacteria and eukaryotes fall into a single NTT subfamily. This clade includes the NTTs of endoparasitic fungi (microsporidians and rozellids), those of primary plastids—which are not closely related to those of modern cyanobacteria—and sequences from Chlamydiae and Alpha-proteobacteria. Diatoms encode multiple NTT genes, including one originating from the primary plastid by secondary endosymbiosis and others obtained from independent horizontal transfer events from bacteria. The NTT module also occurs in combination with other functional domains. NTT-HEAT domain fusions are monophyletic, likely arising once during bacterial evolution, and are widely distributed in bacteria, including in cyanobacteria. The acquisition of an NTT-HEAT fusion protein by horizontal transfer was ancestral in the oomycetes. The tree was inferred under the LG + C60 model in IQ-Tree (Nguyen et al. 2015). See supplementary fig. S1, Supplementary Material online for a fully detailed phylogenetic tree with species names and bootstrap support values.

NTTs have previously been described in intracellular bacteria such as *Chlamydia* and *Rickettsia*, intracellular eukaryotic parasites like Microsporidia and the related endoparasitic rozellids, as well as in plant and diatom plastids (Tjaden et al. 1998; Audia and Winkler 2006; Tsaousis et al. 2008; Ast et al. 2009; James et al. 2013; Heinz et al. 2014). These findings have motivated the view that NTTs are an important hallmark of intracellular parasitism because they function as the main suppliers of energy and nucleotide triphosphates for parasite growth and replication (Dean et al. 2014; Yeoh et al. 2015; Bou Khalil et al. 2016). By contrast, our analysis demonstrates that NTT genes are also broadly distributed among free-living bacteria and non-photosynthetic eukaryotes (fig. 2). We failed to identify any NTT homologues on sequenced archaeal genomes. Our analysis supports a monophyletic grouping of all but three functionally characterized NTTs (fig. 2; bootstrap support 100), comprising sequences from the intracellular pathogens *Chlamydia*, *Rickettsia*, and Microsporidia, and the plastids of plants and diatoms with their diverse substrate specificities and different transport modes. Thus, experimental work to date has investigated a surprisingly small fraction of NTT diversity.

Our analyses identified the first examples of NTT genes in the the rhizarian parasite *Plasmodiophora brassicae*, which causes clubroot disease (Schwelm et al. 2015). The two *Plasmodiophora* NTTs group at the base of the previously characterised (Heinz et al. 2014) fungal (Microsporidia/*Rozella*) NTT clade with high bootstrap support (fig. 2, bootstrap support 92). At face value, this finding suggests either that rhizarians or endoparasitic fungi acquired an NTT from the same bacterial source by horizontal transfer, or else that this NTT gene was transferred between eukaryotes after a single acquisition. Since the position of the endoparasitic fungi was poorly resolved in the full analysis (bootstrap support 53), we inferred an additional tree focusing just on this NTT subfamily to improve resolution (supplementary fig. S2, Supplementary Material online); this tree agreed in placing the rhizarian NTTs within the endoparasitic fungi with maximal bootstrap support (100). Within the clade of NTTs from photosynthetic eukaryotes (fig. 2), we found NTTs in the photosynthetic chromerid *Vitrella brassicaformis*—the first known alveolate with NTTs. Another interesting gene from this clade is an NTT from the Haptophyte *Emiliana huxleyi*, which is C-terminally fused to a predicted S-adenosylmethionine (SAM) dependent methyltransferase (fig. 1). Although no SAM-transporting NTT has been reported, fusion of this enzyme to the transport protein could point to its substrate specificity. To date, SAM transporters from a family unrelated to NTTs have been identified in Chlamydia, Rickettsia, and Bacteroidetes (Tucker et al. 2003; Binet et al. 2011; Haferkamp et al. 2013).

The single characterized bacterial NTT from outside this clade is NTT4 transporter of *Protochlamydia amoebophila* (*Pam*NTT4) which, like some canonical NTTs (Fisher et al. 2013), transports NAD (Haferkamp et al. 2004), and clusters separately from the other *P* *.* *amoebophila* proteins. This transporter groups with a variety of other novel uncharacterized bacterial NTTs, suggesting that these *Pam*NTT4-like proteins (fig. 2, supplementary fig. S1, Supplementary Material online) might also be involved in NAD transport. This clade also contains NTTs from Viridiplantae, Haptophytes, and the ichthyosporean *Capsaspora owczarzaki*. One of the plant NTTs (*Arabidopsis thaliana At*NTT3), has been functionally characterized as an ATP transporter (Piecuch 2008). Given that Haptophytes and Icthyosporeans are not closely related to each other, the tree suggests either parallel acquisition from related bacterial donors, or a single acquisition followed by within-eukaryote transfer.

Although cyanobacteria have NTT fusion proteins (see below), and primary plastids are thought to originate from a single endosymbiotic event involving a cyanobacterium, our analyses do not provide any evidence for a cyanobacterial origin of the NTTs functioning in contemporary primary plastids. Instead our phylogeny (fig. 2, supplementary fig. S1, Supplementary Material online) supports a distinct—possibly chlamydial—common origin for the single-domain NTTs of photosynthetic eukaryotes.

**Fig. 3.—:**
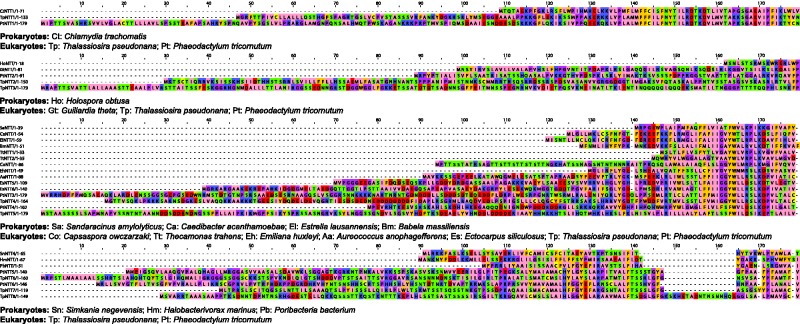
Diatom NTTs gained N-terminal extensions on multiple independent occasions. In addition to the NTT that they share with primary plastids, diatom NTTs appear to have independent bacterial origins. These genes might be involved in energy transfer across the multiple internal membranes of these secondarily photosynthetic eukaryotes, and not all diatom NTTs have *in silico* detectable N-terminal targeting signals (Ast et al. 2009; Chu et al. 2017). Our phylogeny allows direct comparison of Diatom NTTs to their close bacterial relatives and so reveals significant elongations at their N-termini. The role of these sequences in protein targeting to the organelle remains to be evaluated experimentally.

In contrast to the single origin of primary plastid NTTs, we observed multiple independent acquisitions of NTTs in diatoms. Diatoms acquired their plastid through endosymbiosis with a photosynthetic eukaryote, probably a red alga (Armbrust et al. 2004). Consistent with that scenario, diatom NTT1—which has been functionally characterized, and localizes to the plastids of *Thalassiosira pseudonana* and *Phaeodactylum tricornutum* (Ast et al. 2009)—groups with the NTT of primary plastids in our tree (supplementary fig. S1, Supplementary Material online), and was therefore likely obtained by secondary endosymbiosis. Interestingly, diatoms encode more NTT genes than other photosynthetic eukaryotes (e.g. 8 in *Thalassiosira*), and some of these appear to have been acquired independently from bacteria on at least three occasions. Diatom NTT2 also localises to the plastid, but appears to have an independent alpha-proteobacterial origin (supplementary fig. S1, Supplementary Material online), as previously reported by Ast et al. (2009). *Tp*NTT6-8 and *Pt*NTT5-6 group with NTTs from three host-associated bacteria: the sponge symbiont *Poribacterium* (Fieseler et al. 2004), the intracellular bacterial pathogen *Simkania negevensis* NTT4 (Knab et al. 2011), and the fDeltaproteobacterium *Halobacteriovorax marinus* (fig. 2, supplementary fig. S1, Supplementary Material online), a predatory intracellular bacterial pathogen which infects other Gram-negative bacteria (Crossman et al. 2013). *Pt*NTT5 was recently characterized as a purine nucleotide transporter that localises to the endoplasmic reticulum and outermost plasma membrane (Chu et al. 2017) but *S* *.* *negevensis* NTT4 (fig. 2, supplementary fig. S1, Supplementary Material online) failed to transport nucleotides in heterologous host assays (Knab et al. 2011). *Tp*NTT4-5/*Pt*NTT3-4 grouped with sequences from another set of taxonomically diverse intracellular bacteria, including representatives of the *Chlamydiae*, Alpha- and Deltaproteobacteria. The diatom sequences form a monophyletic group with NTTs from other Stramenopiles, the Haptophyte *E* *.* *huxleyi*, the Apusozoan *Thecamonas trahens*, and the eukaryotic snail symbiont *C* *.* *owczarzaki* (Ichthyosporea), suggesting either parallel acquisitions from related bacterial sources or within-eukaryote NTT transfer (supplementary fig. S1, Supplementary Material online). In diatoms, nucleotide biosynthesis occurs in the cytosol—compared with nucleotide synthesis in the plastid in plants. One possibility is that the additional NTT genes in diatoms might be required to move ATP across the extra membranes associated with a secondary plastid (Ast et al. 2009; Chu et al. 2017). Consistent with this hypothesis, alignment of these sequences against their closest bacterial relatives reveals that all diatom NTTs possess N-terminal extensions that may represent signal peptides (fig. 3), although the functions of these sequences remain to be determined experimentally.

A Single Ancestral Event Gave Rise to a Broadly Distributed Family of NTT-HEAT Fusion Proteins

Our NTT domain phylogeny resolves all NTT-HEAT fusion proteins as monophyletic (fig. 2, bootstrap support 100), implying a single origin of this multidomain protein during bacterial evolution. Although difficult to resolve definitively, we suggest that the fusion protein represents the derived state, because its constituent components (NTT domain, HEAT repeat, and cNBD domain) all occur independently in other contexts across the tree of life. The broad distribution of NTT fusions in cyanobacteria is particularly interesting because it raises the possibility of a link between NTT transport activity and photosynthesis. The NTT phylogeny (fig. 2) resolves two distinct clades of cyanobacterial NTT fusions: the NTT-HEAT fusion, which is predominantly present in section III, IV and V cyanobacteria, and the NTT-HEAT-cNBD fusion which is limited to cyanobacteria from section I, II and III. These two clades are also recovered in phylogenies of the HEAT (supplementary fig. S3, Supplementary Material online) and cNBD (supplementary fig. S4, Supplementary Material online) domains. At present, cyanobacterial NTT fusions are annotated in the databases as PBS lyases (PF03130), but none have been functionally characterized. Cyanobacteria possess internal membranes (thylakoids) that harbor proteins involved in photosynthesis; one potential role for NTT fusions could be ATP transfer across these membranes. While it is tempting to speculate that these genes might be involved in photosynthesis, the presence of NTT fusions in other bacteria suggests that this is not their exclusive function. Another possibility is that NTT fusions might be involved in multicellular development, which is found in sections III–V cyanobacteria. In particular, cellular differentiation in sections IV and V cyanobacteria often involves the formation of a heterocyst, a non-photosynthetic cell type that is specialised for nitrogen fixation and which requires ATP input. If expressed on the cyanobacterial cell surface, NTT fusions could potentially facilitate this ATP supply to heterocysts. More generally, ATP exchange might also be a social feature to provide energy to cells that have a lower exposure to sunlight. Finally, NTT fusions in intracellular cyanobacteria could mediate ATP exchange between bacterium and host cell, as occurs in modern plastids. This could be the case for unicellular section I symbionts like *Prochloron didemni* (Schmidt et al. 2005), which is the only section I cyanobacterium grouping in the NTT-HEAT clade of sections III–V, or “multicellular” section IV endosymbionts (Hilton et al. 2013).

The human pathogen *Chlamydia trachomatis* possesses a single NTT-HEAT fusion gene. Despite the detailed functional characterization of two of the single-domain NTT proteins in this organism (Tjaden et al. 1999; Fisher et al. 2013), the NTT fusion remains uncharacterized, and is annotated as a hypothetical protein. The NTT-HEAT fusion is conserved across Chlamydiaceae and *Estrella lausannensis*—a pathogen of amoebae, that can grow in human macrophages (Bertelli et al. 2015)—suggesting functional importance. This would be an excellent candidate for future experimental investigation, not only to better understand the pathobiology of *C. trachomatis* but also because of the role of NTTs as potential drug targets.

Oomycetes are important parasites, including *Phytophthora infestans* (Haas et al. 2009), implicated in the Irish potato famine. Other species can cause life-threatening infections in animals and humans (Mendoza et al. 1993). Uniquely amongst eukaryotes, oomycetes possess fused NTT-HEAT-cNBD proteins and contain a duplicated cNBD domain (fig. 1). In our single-domain NTT tree (fig. 2), the oomycete genes group with a deltaproteobacterial lineage with poor bootstrap support (47). To investigate further, and given that our tree suggests that the NTT fusion is monophyletic (fig. 2), we inferred a tree of the multidomain NTT-HEAT-cNBD fusion proteins, including those from oomycetes and related bacterial lineages (fig. S5, Supplementary Material online). In this tree, the oomycete lineage falls within a clade of sequences from Deltaproteobacteria, Bacilli, and Marinimicrobia with modest support (87). The single sequence from Marinimicrobia is weakly (72 bootstrap support) resolved as the closest relative of the oomycete sequences (supplementary fig. S5, Supplementary Material online), although the neighbouring Bacilli sequences uniquely share the cNBD duplication with oomycetes, and therefore also represent good candidates for the closest living relatives of the donor lineage.

In all of these analyses, oomycete NTT-HEAT-cNBD proteins form a clade with maximal bootstrap support (100) and are encoded on the genomes of species that infect both plants and animals (supplementary table S1, Supplementary Material online), suggesting that acquisition of the NTT fusion protein was an early event in the evolutionary history of the group. Although some published oomycete genomes lack annotated NTTs, we detected sequences with high similarity to annotated oomycete NTT genes using TBLASTN searches in all cases. Interestingly, transcriptome data for the oomycete *Pythium ultimum* (Lévesque et al. 2010) indicate that the NTT fusion gene is expressed, at least in this species. The transcriptome data also revealed the presence of 13 introns in this NTT gene, which may explain why they were missed during some oomycete genome annotation projects (supplementary table S1, Supplementary Material online).

Oomycete NTTs do not share recent common ancestry with those of other stramenopiles, and—as modern oomycetes do not have plastids (Tyler et al. 2006; Jiang and Tyler 2012)—one exciting possibility is that their NTTs could be surface-located and involved in host-parasite interactions, although this hypothesis requires experimental validation. Regardless of subcellular localization, the conservation of these NTTs among oomycetes suggests that they carry out an important function for the pathogens, and the absence of these proteins in the hosts could make them promising drug targets.

We identified one case in which an NTT fusion has reverted to a non-fused state (fig. 2); the resulting NTT domain is apparently functional, as it has been retained on the genomes of several bacteria, forming a clade comprising five sequences from Chloroflexi, Deinococcus-Thermus, Actinobacteria, and Cyanobacteria, as well as two in the Haptophyte *Chrysochromulina*. Our analyses suggest that the C-terminal end of NTT fusions is more evolutionary labile, with multiple fusions and fissions between the HEAT and cNBD domains during bacterial evolution (supplementar fig. S4, Supplementary Material online).

## Conclusions

NTTs are used by parasites and symbionts from across the tree of life to obtain energy from their hosts and to support their DNA and RNA metabolism. The independent, lineage-specific expansions we observe in *Rickettsiae* and Microsporidia, together with the retention of a diverse set of NTTs in Diatoms and *Chlamydiae*, underpin the particular importance of NTTs for an intracellular lifestyle ( supplementary fig. S1, Supplementary Material online), and our discovery of fused NTT genes in oomycetes raises intriguing parallels with the roles of these genes in other parasitic eukaryotes. But our study has also revealed that NTTs are distributed much more widely than has previously been appreciated, including in many non-parasitic bacteria and eukaryotes (fig. 2, supplementary fig. S1, Supplementary Material online) that will serve as a guide to future experimental work. Our results identify a new family of NTT fusion proteins as interesting targets for future characterization to understand energy flow in cyanobacteria, as well as the functional diversity of NTT domains as a whole.

## Materials and Methods

All sequences were obtained from NCBI. Iterative BLASTP searches were performed against the non-redundant protein (nr) database using characterised NTTs from *Chlamydiae*, and subsequently newly discovered NTTs as queries. The sequences used in our analyses are included as a supplementary material S1, Supplementary Material online. The NTT domain was extracted from NTT fusion proteins for phylogenetic analysis by alignment against the single-domain NTT genes. Sequences were aligned using MUSCLE (Edgar 2004), poorly conserved regions were detected and removed with TrimAl (Capella-Gutiérrez et al. 2009) and maximum likelihood phylogenetic trees were inferred under the LG + C60 model in IQ-Tree (Nguyen et al. 2015). HEAT and cNBD trees were inferred using the same protocol.

## Supplementary Material

Supplementary data are available at *Genome Biology and Evolution* online.

## Acknowledgments

This study was supported by a European Research Council Advanced Investigator Award (ERC- 2010-AdG-268701) and Wellcome Trust grant (045404) to T.M.E. and a Royal Society University Research Fellowship (UF140626) to T.A.W.

## Supplementary Material

Supplementary Data
