# Supplementary material for: Phylogenetic Diversity of NTT Nucleotide Transport Proteins in Free-Living and Parasitic Bacteria and Eukaryotes
Source: Genome Biol Evol. 2017 Feb 2;9(2):480–7. doi: 10.1093/gbe/evx015 (PMC5381601; doi:10.1093/gbe/evx015)
Supplement: Supplementary Data [file evx015_Supp.zip › Tab_S1_revised.docx]

**Table S1.** Oomycete NTTs in species without annotated protein sequences at NCBI found with TBLASTN. Genes contain introns. Predicted proteins based on transcriptome data from the *Pythium* Genome Database are given below.

| **Species** | **NCBI genome accession** | **Location on contig** |
| --- | --- | --- |
| *Pythium ultimum* | ADOS00000000.1 | ctg_1117875580994  13350 – 18623 |
| *Pythium insidiosum* | BBXB00000000.1 | PINS000020  262835 – 267730 |
| *Pythium vexans* | AKYC00000000.2 | pve_contig_80  429 – 5679 |
| *Hyaloperonospora arabidopsidis* | LLKM00000000.1 | scf_59133_148.contig_1  46438 – 41379 |
| *Pilasporangium apinafurcum* | BCKE00000000.1 | BCKE01000038  99364 – 94112 |
| *Pseudoperonospora cubensis* | AHJF00000000.1 | psc_contig_1639  6428 – 1382 |
| **Species** | ***Pythium* Genome Database annotation** | |
| *Pythium ultimum* | >PYU1_T010880 | |
| *Pythium arrhenomanes* | AED:0.0298697934183441 | |
| *Pythium irregulare* | AED:0.045801551821592 | |
| *Pythium iwayamai* | AED:0.043559265742948 | |
| *Pythium vexans* | AED:0.0454697958617588 | |
