# Supplementary figures and images for: Phylogenetic Diversity of NTT Nucleotide Transport Proteins in Free-Living and Parasitic Bacteria and Eukaryotes
Source: Genome Biol Evol. 2017 Feb 2;9(2):480–7. doi: 10.1093/gbe/evx015 (PMC5381601; doi:10.1093/gbe/evx015)

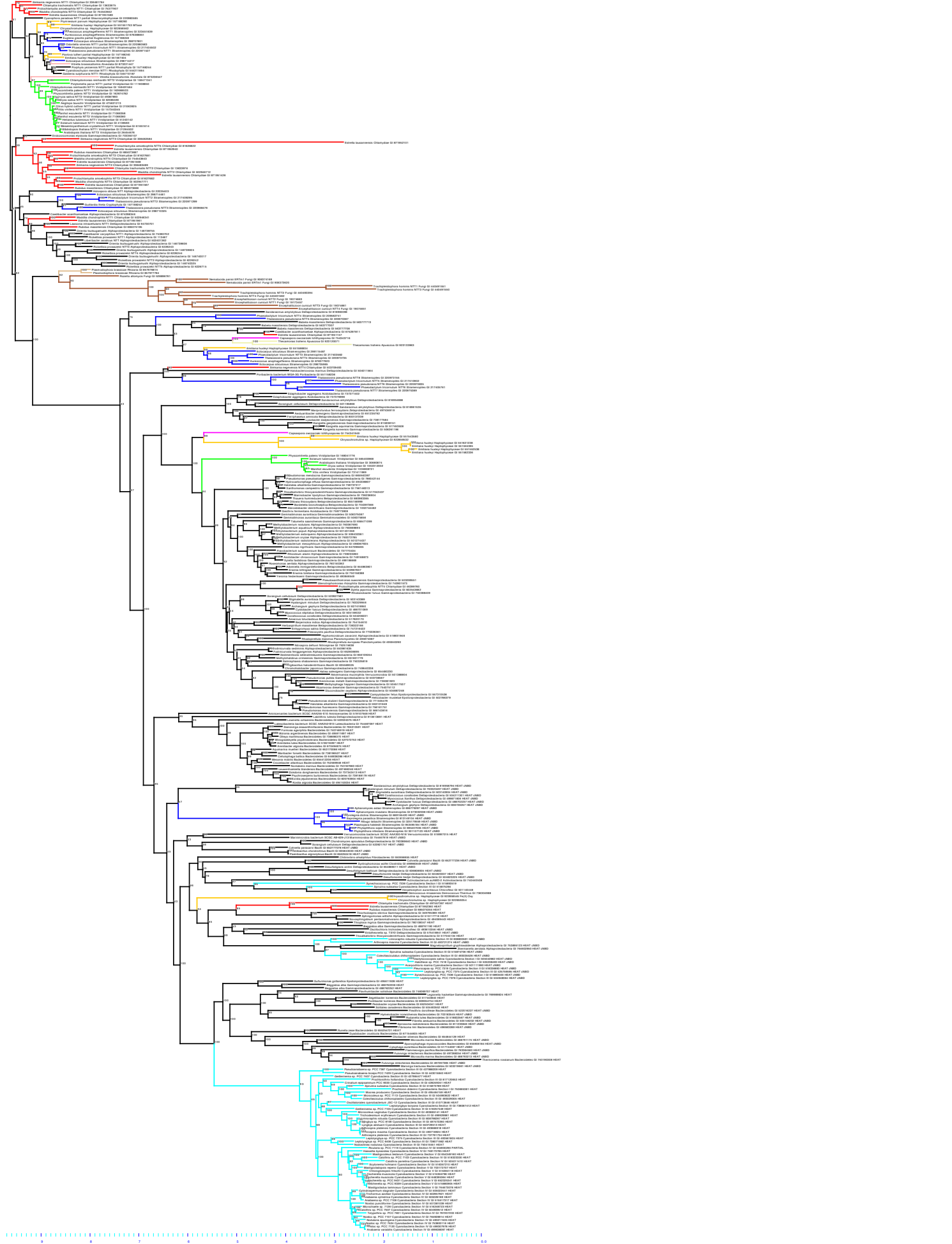

Supplement: Supplementary Data [file evx015_Supp.zip › Fig. S1.pdf]

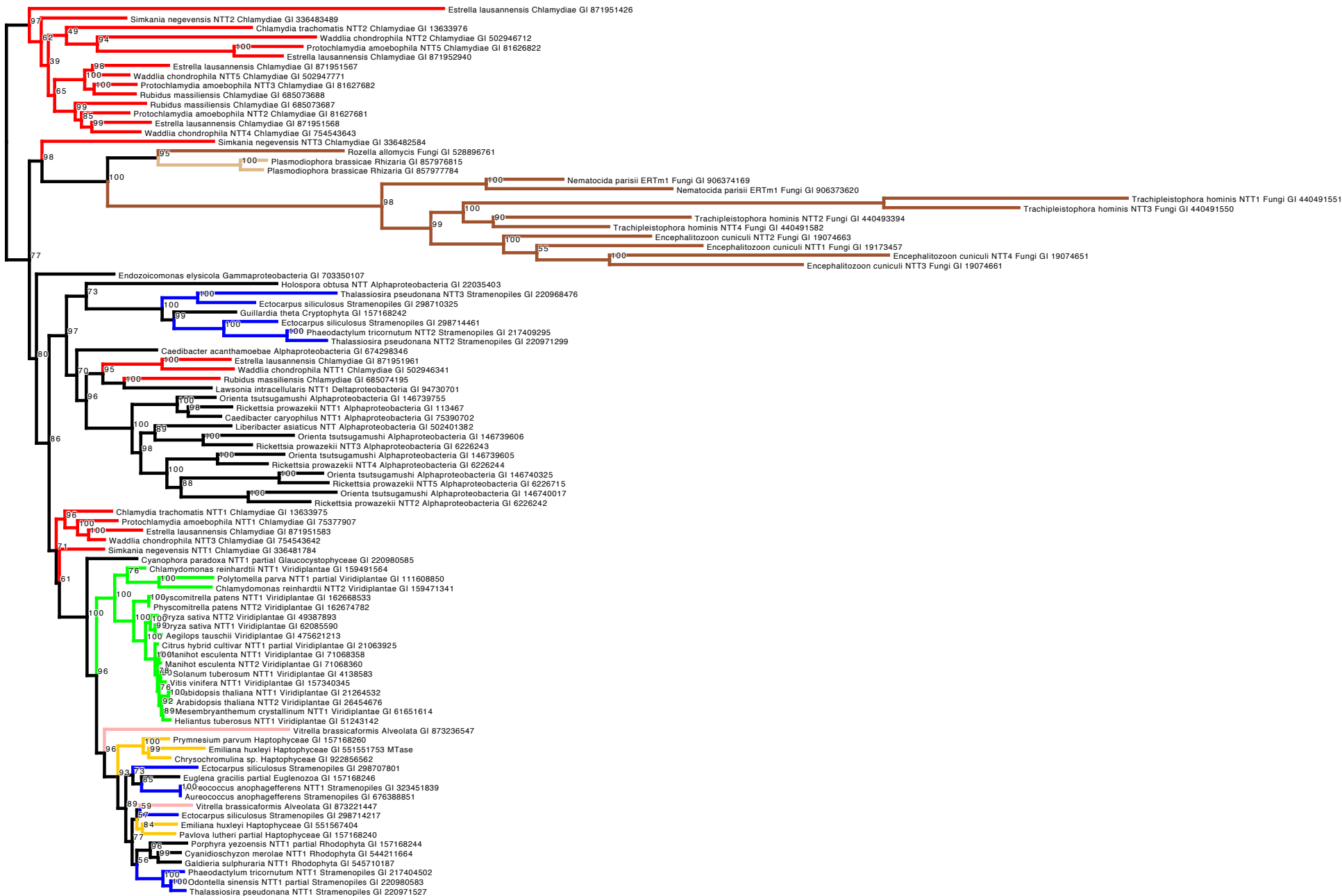

12 11 10 9 8 7 6 5 4 3 2 1 0.0

Supplement: Supplementary Data [file evx015_Supp.zip › Fig. S2.pdf]

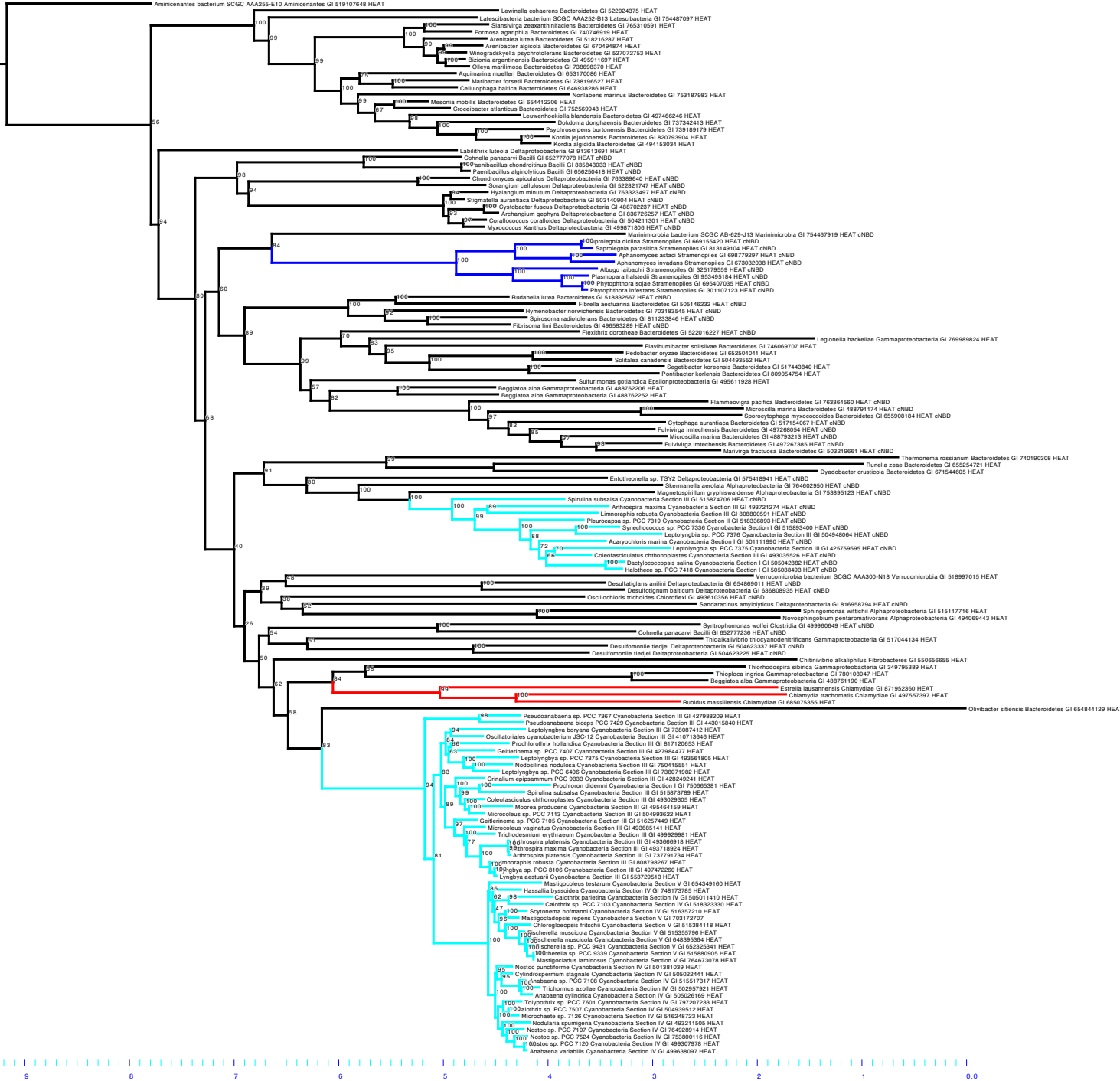

Supplement: Supplementary Data [file evx015_Supp.zip › Fig. S3.pdf]

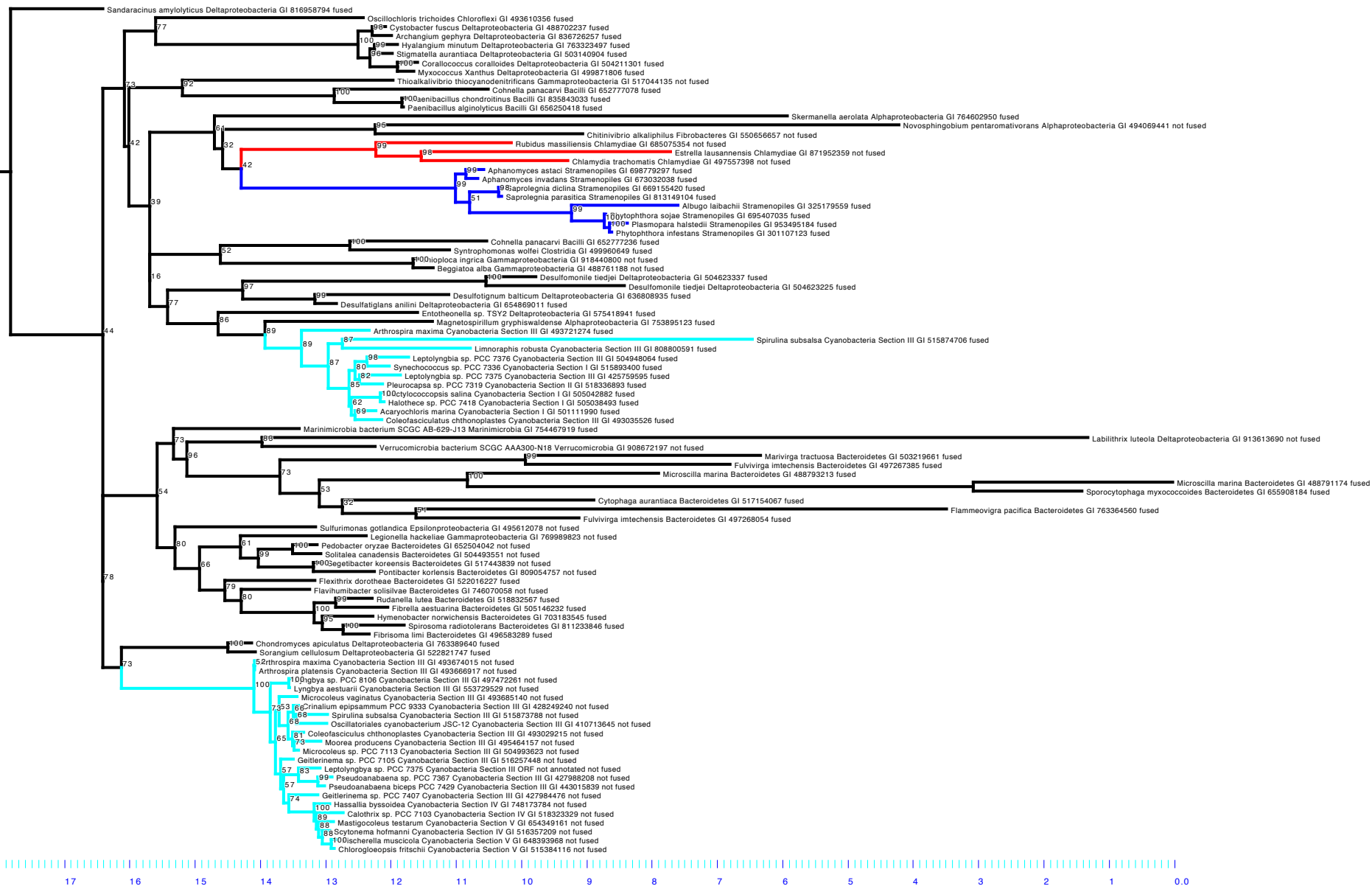

Supplement: Supplementary Data [file evx015_Supp.zip › Fig. S4.pdf]

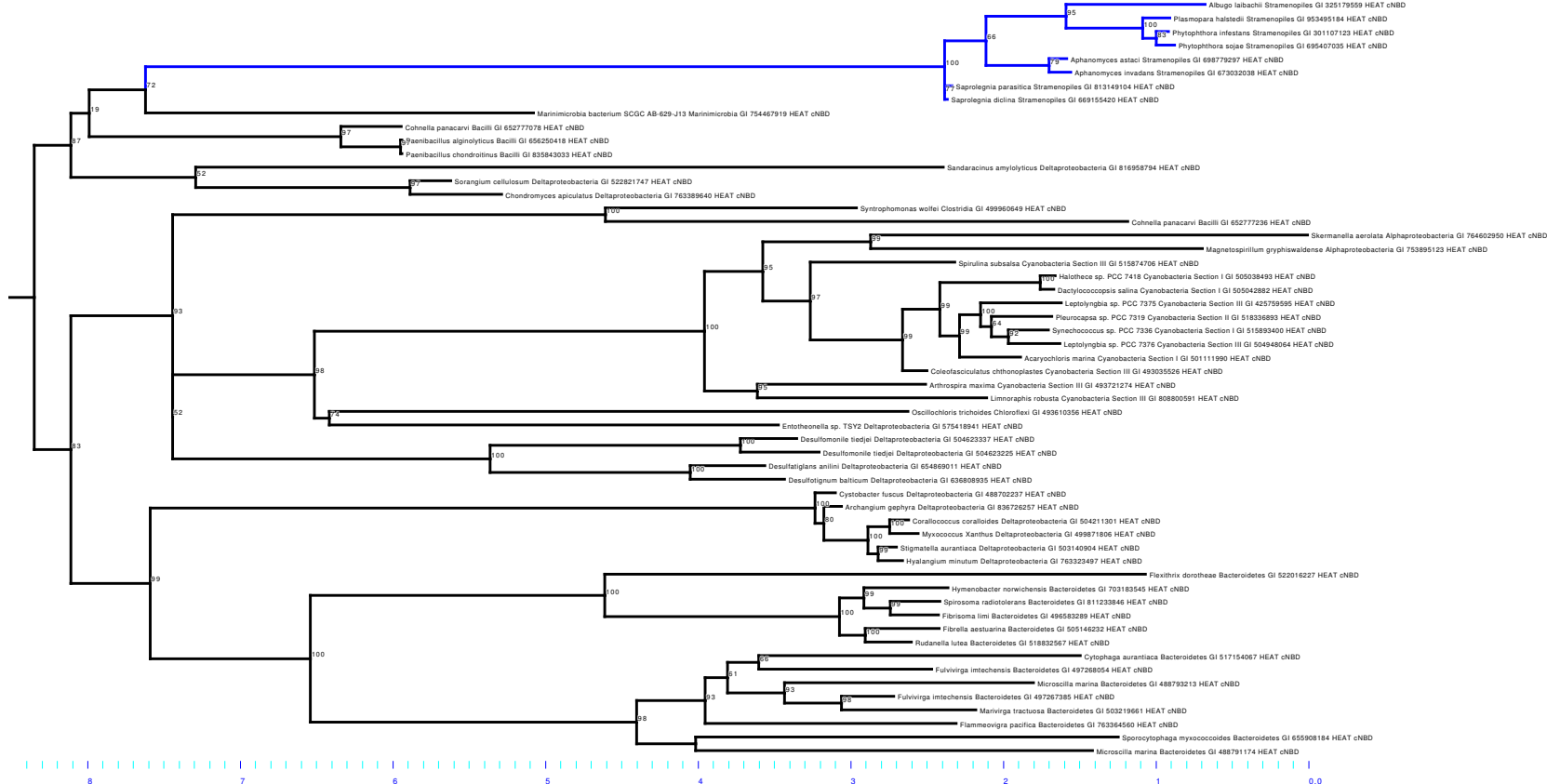

Supplement: Supplementary Data [file evx015_Supp.zip › Fig. S5.pdf]
